# The complete mitochondrial genome of *Pethia padamya* (Actinopteri, Cyprinidae)

**DOI:** 10.1080/23802359.2023.2186724

**Published:** 2023-03-25

**Authors:** Yang Pan, Xiaoyan Xiang, Yuewei Tong, Shubao Hu, Duoqi Zhou, Ganlin Wu, Yifeng Qin

**Affiliations:** a College of Life Science, Key Laboratory of Biodiversity and Ecology Conservation in Southwest Anhui, Anqing Normal University, Anqing, China; b Ministry of Ecology and Environment, Nanjing Institute of Environmental Sciences, Nanjing, China

**Keywords:** Cypriniformes, mitogenome, phylogenetic, Smiliogastrinae

## Abstract

*Pethia padamya* (Kullander and Britz, 2008) is a freshwater fish distributed in the Mekong River basin of Thailand. It has beautiful colors and can be used as an ornamental fish. The complete mitochondrial genome of *P. padamya* was determined using next-generation sequencing technology and its characteristics were analyzed. The mitochondrial genome is a closed circular molecule comprising 16,792 bp, including 13 protein-coding genes, 22 tRNA genes, two rRNA genes, and a major non-coding region. The overall base composition of the mitochondrial genome is 32.47% A, 25.39% C, 26.08% T, and 16.06% G, with a high A + T bias of 58.55%. Phylogenetic analysis revealed *P. padamya* as a sister group of *Pethia conchonius*+(*Pethia ticto*+*Pethia cumingii*) and *Pethia gelius* with maximal support, providing support for the monophyly of the genus *Pethia* based on concatenated nucleotide sequences. The results of this study proved the monophyly of the genus *Pethia*. These data for the first time provide information on the complete mitochondrial genome of *P. padamya* and can contribute to further studies on the biodiversity and management of *P. padamya*.

## Introduction

**Figure 1.**
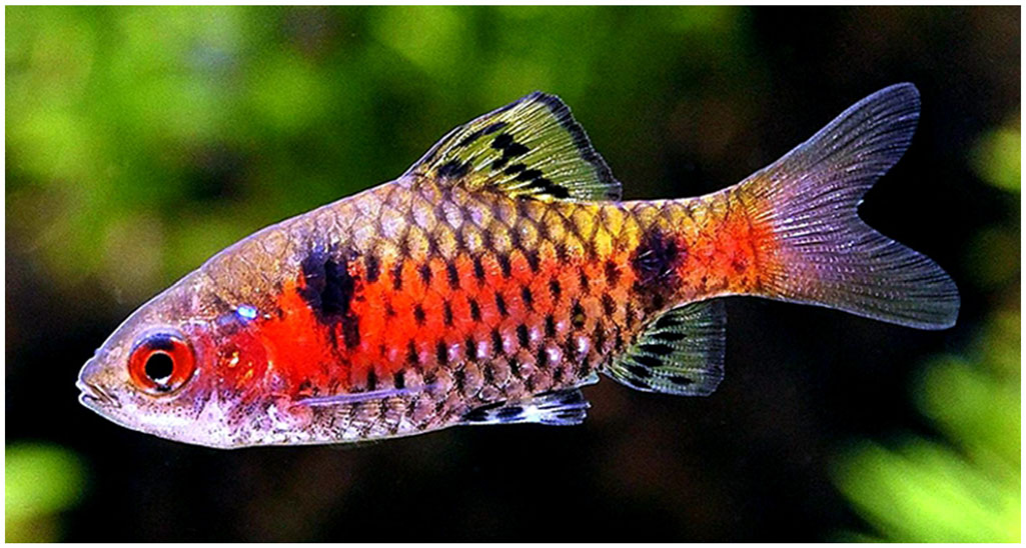
Photograph of *Pethia padamya* (Kullander and Britz 2008). This photograph was taken by the first author (Yang Pan).

*Pethia padamya* (Kullander and Britz 2008) (Figure 1) is a freshwater fish belonging to the order Cypriniformes, family Cyprinidae, and subfamily Smiliogastrinae. *P. padamya* is distributed in the Mekong River Basin in Thailand. It has beautiful colors and can be used as an ornamental fish. It prefers areas where the water is static or submerged areas where the water is covered with aquatic vegetation. The individual fishes prefer to live in groups and have red eyes. When the male fish is colored, a red edge runs from rear the edge of the operculum to the tail. Each fin has a black line, the dorsal fin is yellow, and each scale has a metal reflection (Pethiyagoda et al. 2012). *P. padamya* feeds on small crustaceans, insects, and aquatic plants and does not consume any artificial feed in an artificial environment (Dishma and Vishwanath 2013). The complete mitochondrial genome of *P. padamya* has not been reported. It is thus for the first time reported herein, contributing to further studies on the biodiversity and management of *P. padamya*.

## Materials

The specimens (*P. padamya*) were collected from Kangxi River Flower, Bird, Fish, and Insect Trading Market (30°30′34.02″N, 117°3′15.82″E), No. 245, Huxin South Road, Yingjiang District, Anqing City, Anhui Province, in May 2022, and deposited at the College of Life Science (30°30′52.84″N, 117°2′56.32″E), Anqing Normal University, Anhui Province, China (https://www.aqnu.edu.cn/, Yang Pan, E-mail: panyang2929@163.com) under voucher number Ppadamya-202208-01. This study was approved by the Institutional Animal Care and Use Committee of the Anqing Normal University.

## Methods

The caudal fin was collected, and total DNA was extracted using a columnar animal DNA extraction kit (Sun et al. 2022). Sequencing was performed using the Illumina Novaseq 6000 platform (San Diego, CA). Spades v3.11.1 (Bankevich et al. 2012) and NOVOPlasty (Dierckxsens et al. 2017) were used to assemble the mitochondrial gene using *Pethia conchonius* (AB863607) (Xu et al. 2015) as the reference, and a circular contig of the *P. padamya* mitochondrial gene was obtained. The resulting contig consensus sequence was annotated using MITOS (Bernt et al. 2013) and MitoAnnotator (Iwasaki et al. 2013), and the gene boundary was manually proofread.

To analyze the relative phylogenetic position of *P. padamya* in the subfamily Smiliogastrinae, relevant gene sequences (17 species of Smiliogastrinae and one outgroup *Corydoras aeneus* MZ571336) (Sun et al. 2022) were downloaded from GenBank. Using the tandem sequence set of 13 PCGs and two rRNAs, MrBayes v3.2.6 (Ronquist et al. 2012) was used to construct the Bayesian inference (BI) tree. ModelFinder (Kalyaanamoorthy et al. 2017) was used to select the best-fit partition model (edge-unlinked) using the BIC criterion. BI phylogenies were inferred under two parallel runs of 10,000,000 generations, in which the initial 25% of the sampled data was discarded as burn-in. The MitoFish (http://mitofish.aori.u-tokyo.ac.jp/) online tool was used to generate the circular mitogenome maps.

## Results

**Figure 2.**
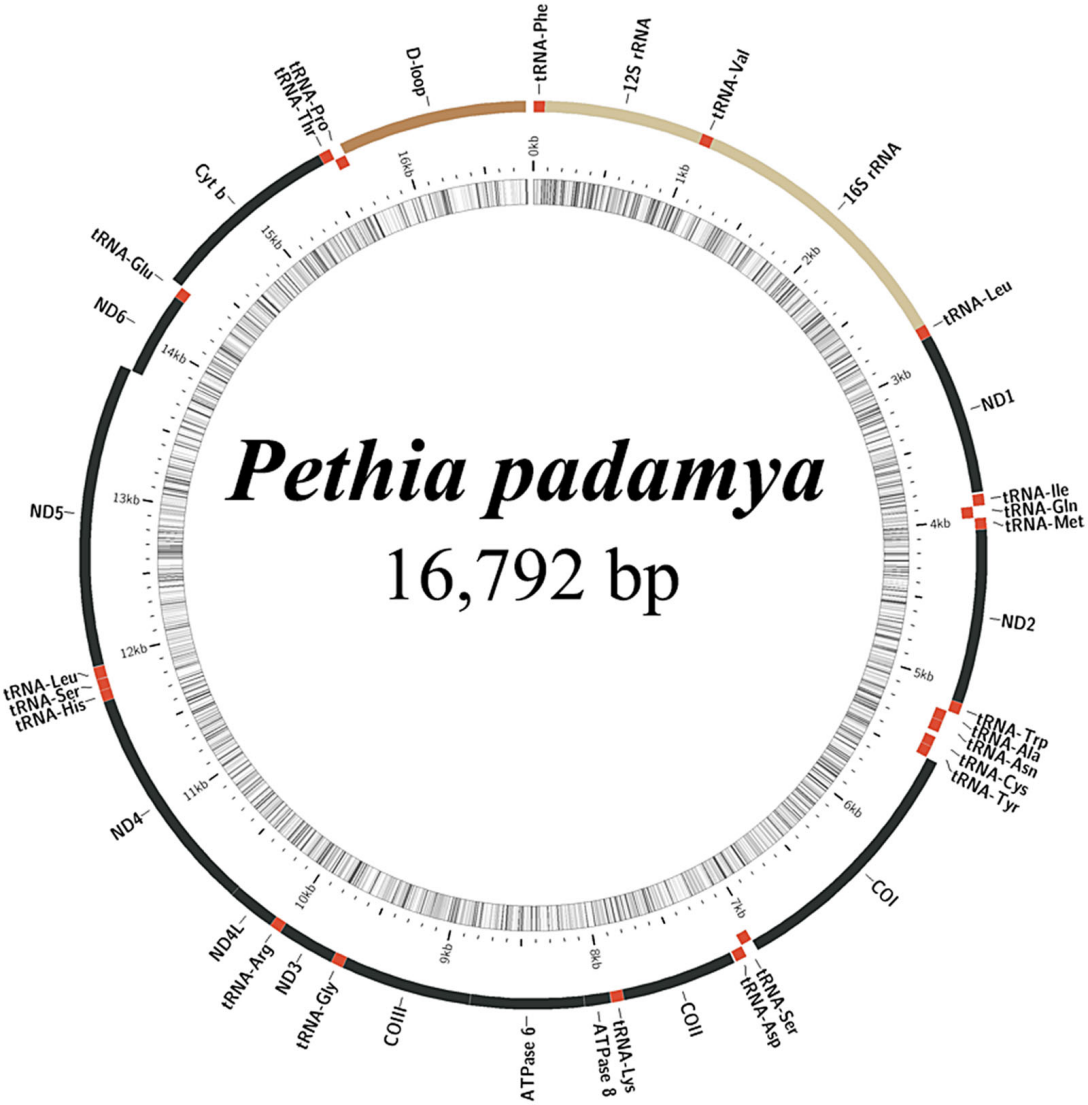
Gene map of the mitochondrial genome of *Pethia padamya.*

Sequencing and assembly analyses showed that the complete mitochondrial genome of *P. padamya* is a closed circular molecule with a total length of 16,792 bp, including 13 protein-coding genes, 22 tRNA genes, two rRNA genes, and a major non-coding region (Figure 2, accession number ON864408). The gene structure and sequence were consistent with those of the mitochondrial genomes of Cyprinidae fish. tRNA-Gln, tRNA-Ala, tRNA-Asn, tRNA-Cys, tRNA-Tyr, tRNA-Ser, tRNA-Glu, tRNA-Pro, and ND6 are encoded on the light chain, and other genes are encoded on the heavy chain. DNASTAR (Madison, WI) was used to determine the base composition of the complete mitochondrial genome. The A + T content of the mitochondrial genome was 58.55%, which is consistent with the results for other vertebrates (Sun et al. 2020).

**Figure 3.**
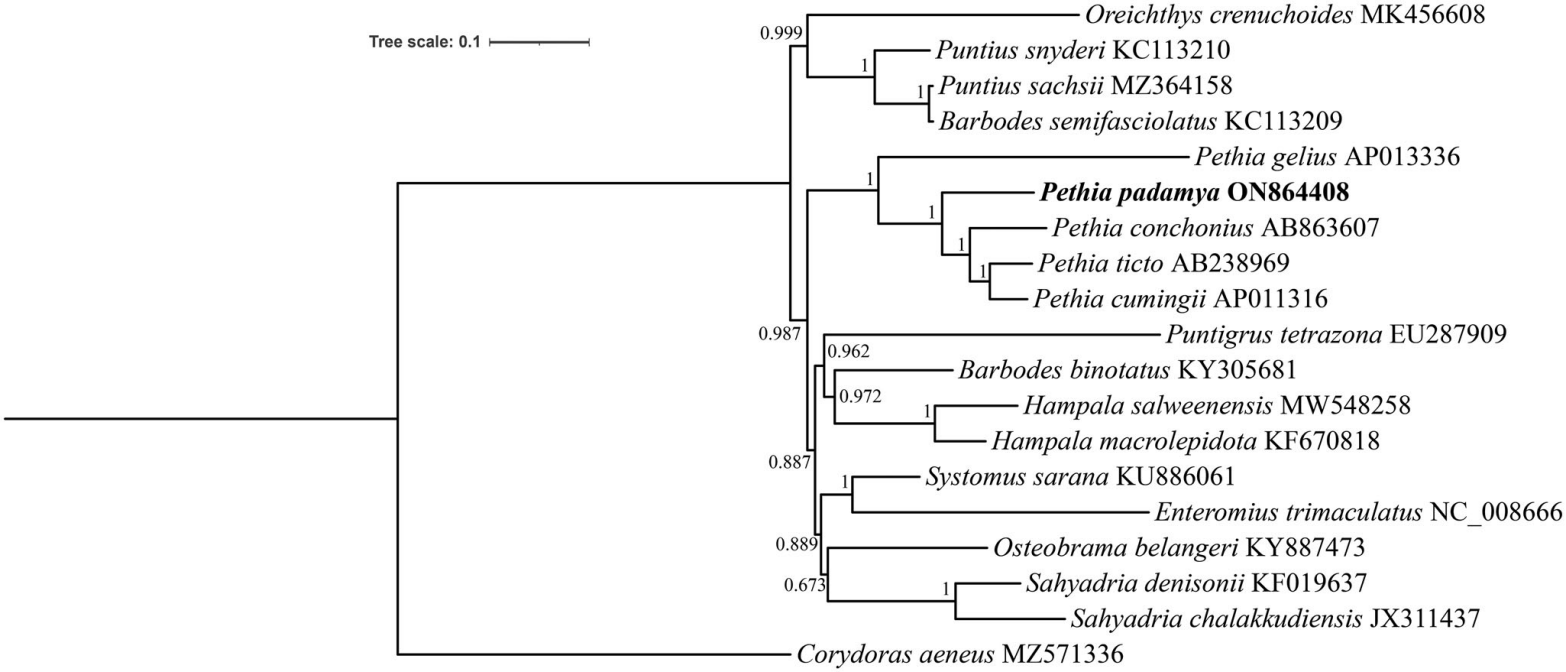
Bayesian inference tree based on the concatenated sequence of 13 protein-coding genes and two rRNAs. The *Pethia padamya* genome is marked in bold font.

To validate the phylogenetic position of *P. padamya* with *Pethia* species, a phylogenetic tree was reconstructed based on concatenated sequences of 13 protein-coding genes and two rRNAs using BI analysis. As shown in the phylogenetic tree (Figure 3), *P. padamya* was identified as the sister group of *Pethia conchonius* + (*Pethia ticto* + *Pethia cumingii*) and *Pethia gelius* with maximal support, validating the close relationship among the five species of the genus *Pethia*.

## Discussion and conclusions

The complete mitochondrial genome of *P. padamya* was determined using next-generation sequencing technology and its characteristics were analyzed. The mitochondrial genome is a closed circular molecule comprising 16,792 bp, including 13 protein-coding genes, 22 tRNA genes, two rRNA genes, and a major non-coding region. These phylogenetic results agree with those of the traditional morphological classification (Katwate et al. 2014; Sudasinghe et al. 2021). Further, the results of this study proved the monophyly of the genus *Pethia* (Dishma and Vishwanath 2013). These data for the first time provide information on the complete mitochondrial genome of *P. padamya* and can contribute to further studies on the biodiversity and management of *P. padamya*.

## Data Availability

The data that support the finding of this paper are available in GenBank of NCBI at http://www.ncbi.nlm.nih.gov/, bio-project, bio-sample, SRA, and mitochondrial genome numbers PRJNA881459, SAMN30903989, SRR21614912, and ON864408, respectively.
